# The impact of migraine prevention on daily activities: a longitudinal and responder analysis from three topiramate placebo-controlled clinical trials

**DOI:** 10.1186/1477-7525-5-56

**Published:** 2007-10-04

**Authors:** Carl Dahlöf, Elizabeth Loder, Merle Diamond, Marcia Rupnow, George Papadopoulos, Lian Mao

**Affiliations:** 1 Gothenburg Migraine Clinic, c/o Läkarhuset, Södra vägen 27, S-41135 Gothenburg, Sweden; 2 Spaulding Rehabilitation Hospital, 125 Nashua Street Boston, MA 02114, USA; 3 Diamond Headache Clinic, 467 Deming Place, Suite 500, Chicago, IL 60614, USA; 4 Ortho-McNeil Janssen Scientific Affairs, LLC, 1125 Trenton-Harbourton Road, Titusville, NJ 08560, USA; 5 Johnson & Johnson Pharmaceutical Services, LLC, 700 Route 202 South, Raritan, NJ 08530, USA

## Abstract

**Background:**

Topiramate is approved for the prophylaxis (prevention) of migraine headache in adults. The most common adverse events in the three pivotal, randomized, double-blind, placebo-controlled trials were paresthesia, fatigue, cognitive impairment, anorexia, nausea, and taste alteration. In these trials, topiramate 100 mg/d significantly improved Migraine-Specific Questionnaire (MSQ) scores versus placebo (p < 0.001). The MSQ measures how much migraine limits/interrupts daily performance. Pooled analyses of pivotal trial data were conducted to further assess how topiramate 100 mg/d affects daily activities and patient functioning.

**Methods:**

Mean MSQ and Medical Outcome Study Short Form 36 (SF-36) change scores (baseline to each double-blind assessment point) were calculated for pooled intent-to-treat (ITT) patients. Additionally, pooled ITT patients receiving topiramate 100 mg/d or placebo were combined and divided into two responder groups according to percent reduction in monthly migraine frequency: < 50% responders or ≥ 50% responders. Between-group differences were assessed using analysis of covariance.

**Results:**

Of 756 patients (mean age 39.8 years, 86% female), 384 received topiramate 100 mg/d and 372 placebo. Topiramate significantly improved all three MSQ domains throughout the double-blind phase versus placebo (p = 0.024 [week 8], p < 0.001 [weeks 16 and 26] for role prevention; p < 0.001 for role restriction and emotional function [all time points]). Topiramate 100 mg/d significantly improved SF-36 physical component scores (PCS) throughout the double-blind phase versus placebo (p < 0.001, all time points) and significantly improved mental component scores (MCS) at week 26 (p = 0.043). The greatest topiramate-associated improvements on SF-36 subscales were seen for bodily pain and general health perceptions (p < 0.05; weeks 8, 16, and 26), and physical functioning, vitality, role-physical, and social functioning (p < 0.05; weeks 16 and 26). Significantly greater improvements in all three MSQ domains, as well as the PCS and MCS of SF-36, were observed for ≥ 50% responders versus < 50% responders (p < 0.001). Significantly greater percentages of topiramate-treated patients were ≥ 50% responders versus placebo (46% versus 23%; p < 0.001).

**Conclusion:**

Topiramate 100 mg/d significantly improved daily activities and patient functioning at all time points throughout the double-blind phase. Daily function and health status significantly improved for those achieving a ≥ 50% migraine frequency reduction.

## Introduction

Migraine is a significant detriment to daily functioning and productivity during, but also to a certain extent before and after, attacks. Migraine preventive therapy should improve negative, disease-related outcomes, provided that appropriate diagnostic criteria and management guidelines are followed. Although approximately 11.5 million U.S. patients with migraine could benefit from preventive therapy (approximately 40% of all U.S. patients with migraine), only one in five currently receives this type of treatment [1,2].

In three large, randomized, double-blind, placebo-controlled, 6-month, migraine prevention trials [3-6], topiramate 100 mg/d (50 mg bid) was associated with a significant and sustained decrease in mean monthly migraine frequency, observed as early as the first month of therapy. Topiramate 100 mg/d was generally well tolerated and is recommended as the target dose for most patients with migraine [3-6]. Recently, safety and tolerability data for these three trials was pooled and analyzed [6]. The most common adverse events in topiramate-treated patients in these trials were paresthesia (50.5%), fatigue (15.0%), anorexia (14.5%), upper respiratory infection (14.0%), cognitive impairment (13.7%), nausea (13.2%), diarrhea (11.1%), and weight decrease (9.1%). Adverse events were mostly mild to moderate in severity and occurred more frequently during titration to target doses [7]. For a more detailed analysis of safety and tolerability data, the published trials can be reviewed [3-5].

Pooled Migraine-Specific Questionnaire (MSQ) data from the three pivotal trials showed that topiramate 100 mg/d significantly improved each of the three dimensions of MSQ at end point compared with placebo [8]. In addition, analyses of the effects of topiramate migraine preventive therapy on the two activity-related domains of the MSQ and the Medical Outcomes Study Short Form-36 (SF-36), prospectively designated as outcome measures in the North American pivotal trials, were recently published [9,10]. Improvements in patient-reported MSQ outcomes were significantly better for patients receiving topiramate than for those receiving placebo. In addition, improvements in the selected MSQ and SF-36 domains were significantly correlated with the decrease in mean monthly migraine frequency observed with topiramate treatment [9,10].

To further assess the impact of topiramate on daily activities and function over time, results derived from both the MSQ and the generic SF-36 were analyzed over the three time points during the 26-week double-blind phase when the questionnaires were administered. Additionally, we sought to determine the relationship between improvement in these quality of life measures and reductions in migraine frequency.

## Methods

### Study design

The overall study design for patients receiving topiramate 100 mg/d or placebo in the three similar placebo-controlled pivotal trials has been described previously [3-5].

### Patients

Patients were required to have three to 12 migraine periods (one period equals 24 hours with migraine; ie, migraine frequency) and no more than 15 headache days during the 28-day prospective baseline phase. Patients were allowed to continue taking specific agents for acute treatment of migraine, but those who overused acute medications were excluded from the placebo-controlled trials [3,4], except in the trial with propranolol as an active comparator [5]. The intent-to-treat (ITT) population was identified as those who received ≥ 1 post-baseline dose of study medication and contributed efficacy data during the double-blind phase. Demographics for the pooled ITT population are presented in Table 1.

**Table 1:** Baseline demographics for the intent-to-treat populations from the three pivotal migraine prevention trials

**Pivotal Topiramate Trials**									
	**Silberstein 2004 [3]**		**Brandes 2004 [4]**		**Diener 2004 [5]**		**Pooled**		

	Placebo	Topiramate 100 mg/d	Placebo	Topiramate 100 mg/d	Placebo	Topiramate 100 mg/d	Placebo	Topiramate 100 mg/d	P value*

No. of Patients (completers)	115 (69)	125 (83)	114 (63)	120 (63)	143 (99)	139 (94)	372 (231)	384 (240)	
Age, Mean ± SD (yrs)	40.4 ± 11.5	40.6 ± 11.0	38.3 ± 12.0	39.1 ± 12.6	40.4 ± 10.1	39.8 ± 10.9	39.8 ± 11.1	39.8 ± 11.5	0.939
Female, n (%)	103 (90%)	112 (90%)	94 (82%)	109 (91%)	109 (76%)	110 (79%)	306 (82%)	331 (86%)	0.160
Caucasian, n (%)	107 (93%)	117 (94%)	101 (89%)	108 (90%)	127 (89%)	122 (88%)	335 (90%)	347 (90%)	0.965

*p value is from analysis of variance model with treatment and study as fixed factors for age comparison; p values are from Cochran-Mantel-Haenszel general association test controlling for study for gender and race comparisons.

### Outcome measures

MSQ and SF-36 data were collected at baseline and at weeks 8, 16, and 26. End point was defined as the last available post-baseline observation in the double-blind phase. The 14-item MSQ assesses the degree to which migraine limits and interrupts daily performance and is divided into three domains: Role Restriction assesses the degree to which migraine limits performance of daily life, social life, and work (seven questions); Role Prevention assesses the degree to which migraine prevents performance of daily life, social life, and work (four questions); and Emotional Function measures the feeling of frustration and helplessness as a result of migraine (three questions). All three MSQ domains are scored from 0 to 100, with a higher score indicating better functioning [11]. The reliability and validity of the MSQ has been demonstrated in numerous studies [11-13].

The generic SF-36 is a widely used, validated tool that assesses the general impact of medical disorders on multiple domains of patient daily activities and function. It consists of two aggregate summary measures (scales) and eight domain-specific measures (subscales) and is scored from 0 to 100, with a higher score indicating greater function. A change of five points on the SF-36 is generally considered clinically meaningful.

The change in mean MSQ or SF-36 scores from baseline through the double-blind phase was assessed for pooled ITT patients. Baseline values for MSQ and SF-36 are summarized in Table 2. In an additional analysis reflecting International Headache Society clinical trial guidelines, pooled ITT patients on topiramate 100 mg/d or placebo were combined and divided into groups based on whether they had experienced ≥ 50% or < 50% reductions in monthly migraine frequency at the study end point. Changes in MSQ and SF-36 scores from baseline to end point were compared between these responder groups.

**Table 2:** Migraine-Specific Questionnaire (MSQ) and Medical Outcome Study Short Form 36 (SF-36) baseline scores for the pooled intent-to-treat population

	**Baseline Scores (Mean ± SD)**		
	
**MSQ Domains**	**Placebo (n = 372)**	**Topiramate 100 mg/d (n = 384)**	p value*
Role Restriction	51.6 ± 16.2	48.1 ± 17.2	0.008
Role Prevention	68.6 ± 18.9	67.0 ± 19.4	0.285
Emotional Function	57.1 ± 22.7	53.7 ± 24.2	0.087

**SF-36 Domains**	**Baseline Scores (Mean ± SD)**		
	
	**Placebo (n = 372)**	**Topiramate 100 mg/d (n = 384)**	**p-value**

Physical Functioning	82.0 ± 20.1	82.1 ± 19.4	0.920
Bodily Pain	51.3 ± 22.9	51.5 ± 22.4	0.969
Vitality	51.3 ± 19.4	50.8 ± 20.8	0.891
General Health	68.0 ± 19.5	68.8 ± 20.2	0.593
Role-Emotional	69.0 ± 39.3	70.9 ± 37.7	0.486
Role-Physical	44.5 ± 39.9	44.2 ± 40.8	0.959
Mental Health	70.7 ± 17.7	71.4 ± 16.7	0.483
Social Functioning	68.5 ± 22.3	70.0 ± 23.5	0.331
Physical Component Summary	43.1 ± 8.6	42.9 ± 9.1	0.767
Mental Component Summary	46.9 ± 10.6	47.5 ± 10.2	0.396

*p values are from analysis of variance model with treatment and study as fixed factors.

### Statistical analysis

Between-group differences were analyzed using an analysis of covariance model, with treatment and protocol as main effects and respective baseline values as covariates. The responder rate was analyzed using the Cochran-Mantel-Haenszel test adjusting for protocol and analysis center. For these exploratory post hoc analyses, the statistical methodologies used to assess differences between groups were not adjusted for multiplicity. Descriptive statistics were used for the demographic analysis.

## Results

### Demographic and baseline characteristics of patients

Demographics and baseline MSQ and SF-36 scores for the pooled ITT population are presented in Tables 1 and 2. A total of 756 patients given topiramate 100 mg/d (n = 384) or placebo (n = 372) were included in this analysis. The mean (± SD) ages of the patients in these trials ranged from 38.3 (± 12.0) years to 40.6 (± 11.0) years (range, 12 to 70 years). The majority of patients in the three trials were female (≥ 76%) and Caucasian (≥ 88%). The mean (± SD) MSQ scores at baseline (± SD) ranged from 48.1 (± 17.2) to 68.6 (± 18.9). The mean (± SD) SF-36 scores at baseline ranged from 42.9 (± 9.1) to 82.1 (± 19.4). Pooled ITT patients receiving topiramate 100 mg/d or placebo were combined and divided into two response groups < 50% response (n = 492) or ≥ 50% response (n = 262).

### Outcome measures

#### MSQ scores

**Figure 1:**
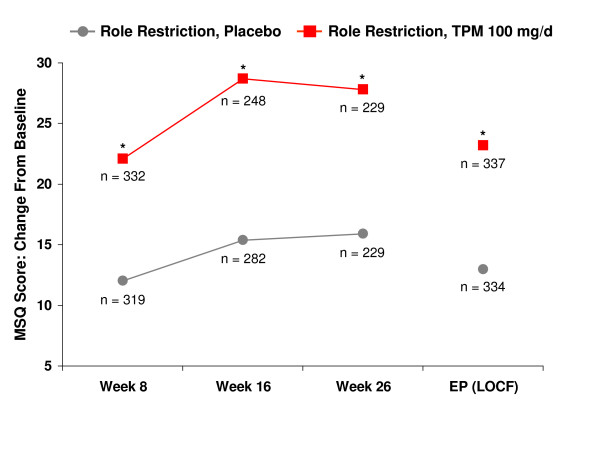
Time course of improvement – Role restriction: mean change from baseline in Migraine-Specific Questionnaire (MSQ) domain scores. *p < 0.001, ^†^p = 0.024 versus placebo. EP = end point; LOCF = last observation carried forward; TPM = topiramate.

**Figure 2:**
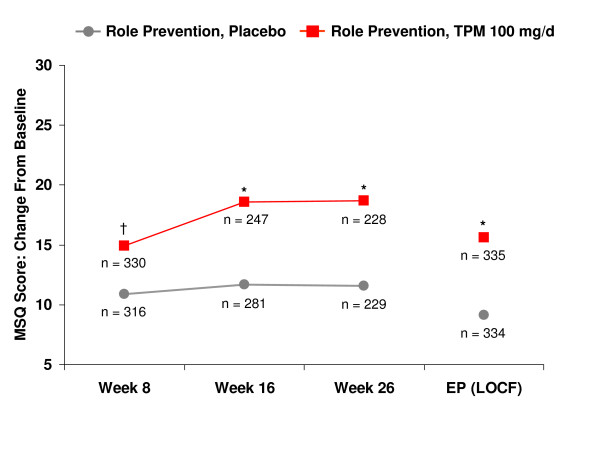
Time course of improvement – Role prevention: mean change from baseline in Migraine-Specific Questionnaire (MSQ) domain scores. *p < 0.001, ^†^p = 0.024 versus placebo. EP = end point; LOCF = last observation carried forward; TPM = topiramate.

**Figure 3:**
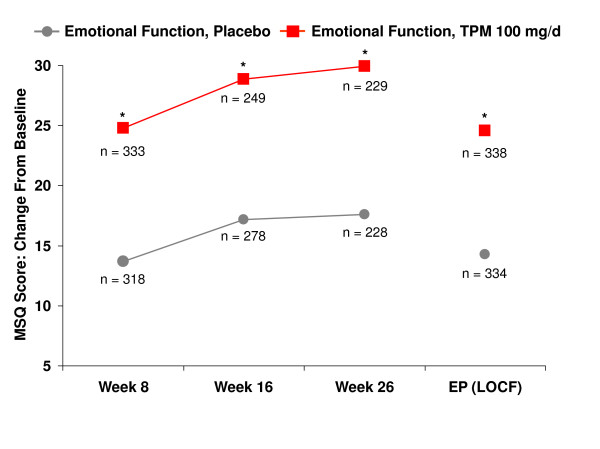
Time course of improvement – Emotional function: mean change from baseline in Migraine-Specific Questionnaire (MSQ) domain scores. *p < 0.001, ^†^p = 0.024 versus placebo. EP = end point; LOCF = last observation carried forward; TPM = topiramate.

Topiramate 100 mg/d significantly improved the mean scores from baseline for all three domains (Role Restriction, Role Prevention, and Emotional Function) of the MSQ at weeks 8, 16, and 26, and at end point compared with placebo (p < 0.001 for all, except Role Prevention p = 0.024 at week 8) (Figures 1, 2, 3).

#### SF-36 scores

**Figure 4:**
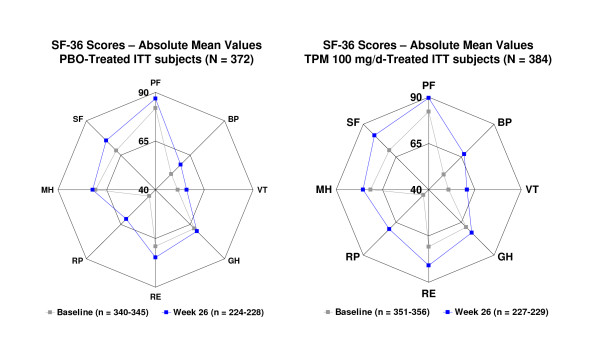
Radar plots of absolute mean Medical Outcome Study Short Form 36 (SF-36) scores at baseline and week 26. PBO = placebo; TPM = topiramate; PF = physical functioning; BP = bodily pain; VT = vitality; GH = general health perceptions; RE = role-emotional; RP = role-physical; MH = mental health; SF = social functioning; ITT = intent-to-treat; TPM = topiramate. All p values versus PBO from baseline to week 26: PF, p = 0.023; BP, p < 0.001; VT, p < 0.001; GH, p = 0.027; RP, p = 0.018); MH, p = 0.034; SF, p = 0.010.

Topiramate treatment resulted in significant improvement compared with placebo at week 26 on all SF-36 subscales except Role-Emotional (Figure 4, Table 3). Topiramate treatment significantly improved the SF-36 physical component scores throughout the double-blind phase compared with placebo (p < 0.001, all time points) and significantly improved the mental component scores at week 26 (p = 0.043). Maximal topiramate-associated improvements on SF-36 subscales were seen for Bodily Pain and General Health Perceptions (p < 0.05; weeks 8, 16, and 26) and Physical Functioning, Vitality, Role-Physical, and Social Functioning (p < 0.05; weeks 16 and 26).

**Table 3:** Change from baseline over time in Medical Outcome Study Short Form 36 (SF-36) domain scores

**SF-36 Domain**	**Treatment Group**	**Week 8**	**Week 16**	**Week 26**	**End Point**
Physical Functioning	Placebo	3.2 ± 0.8	3.2 ± 0.9	5.0 ± 1.0	3.6 ± 0.9
	Topiramate	4.8 ± 0.8	6.4 ± 0.9	8.3 ± 1.0	5.3 ± 0.8
		NS	p = 0.025	p = 0.023	NS
Bodily Pain	Placebo	6.2 ± 1.2	6.7 ± 1.3	6.7 ± 1.4	4.6 ± 1.2
	Topiramate	12.0 ± 1.1	14.3 ± 1.4	14.5 ± 1.4	11.5 ± 1.2
		p < 0.001	p < 0.001	p < 0.001	p < 0.001
Vitality	Placebo	1.9 ± 1.0	3.1 ± 1.0	3.9 ± 1.2	1.8 ± 1.0
	Topiramate	3.8 ± 1.0	7.9 ± 1.1	10.1 ± 1.2	5.2 ± 1.0
		NS	p = 0.008	p < 0.001	p = 0.035
General Health	Placebo	1.0 ± 0.7	0.4 ± 0.8	1.6 ± 0.9	0.8 ± 0.8
	Topiramate	3.4 ± 0.7	4.3 ± 0.9	4.5 ± 0.9	2.2 ± 0.8
		p = 0.009	p < 0.001	p = 0.027	NS
Role- Emotional	Placebo	4.5 ± 2.0	4.1 ± 2.1	6.5 ± 2.3	3.0 ± 2.0
	Topiramate	2.2 ± 2.0	8.0 ± 2.2	9.3 ± 2.3	2.3 ± 2.0
		NS	NS	NS	NS
Role- Physical	Placebo	14.4 ± 2.2	15.0 ± 2.2	15.9 ± 2.5	12.0 ± 2.1
	Topiramate	19.3 ± 2.2	26.6 ± 2.4	24.1 ± 2.5	17.9 ± 2.1
		NS	p < 0.001	p = 0.018	NS
Mental Health	Placebo	-0.2 ± 0.9	0.8 ± 0.8	0.6 ± 1.0	-0.2 ± 0.9
	Topiramate	-1.5 ± 0.9	2.9 ± 0.9	3.2 ± 1.0	-0.5 ± 0.9
		NS	NS	p = 0.034	NS
Social Functioning	Placebo	5.3 ± 1.2	7.8 ± 1.2	6.8 ± 1.3	4.8 ± 1.2
	Topiramate	4.8 ± 1.2	8.5 ± 1.3	10.8 ± 1.3	4.8 ± 1.2
		NS	NS	p = 0.010	NS
Physical Component Summary	Placebo	2.8 ± 0.4	2.8 ± 0.4	3.2 ± 0.5	2.5 ± 0.4
	Topiramate	5.1 ± 0.4	5.7 ± 0.5	5.7 ± 0.5	4.7 ± 0.4
		p < 0.001	p < 0.001	p < 0.001	p < 0.001
Mental Component Summary	Placebo	0.3 ± 0.6	0.8 ± 0.5	0.9 ± 0.6	0.1 ± 0.5
	Topiramate	-0.6 ± 0.6	1.5 ± 0.6	2.2 ± 0.6	-0.2 ± 0.5
		NS	NS	p = 0.043	NS

#### Responder analysis

**Figure 5:**
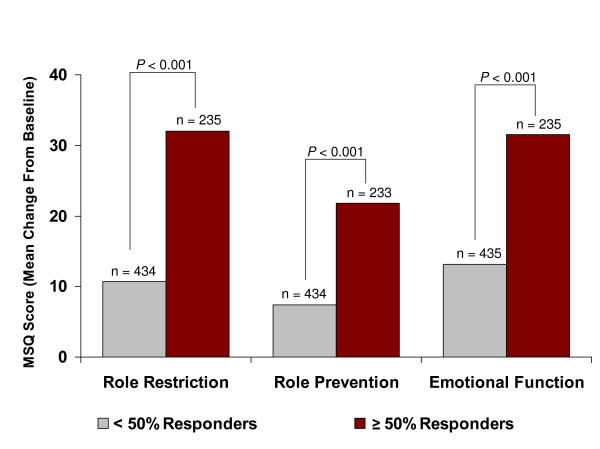
Migraine-Specific Questionnaire (MSQ) domain scores at end point (last observation carried forward) for pooled intent-to-treat subjects with ≥ 50% or < 50% reductions in monthly migraine frequency.

**Figure 6:**
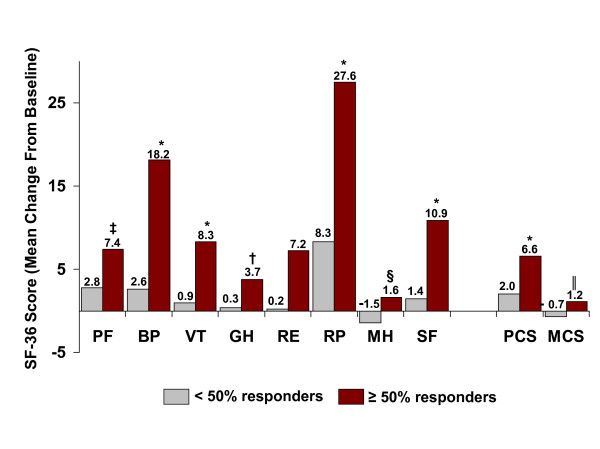
Medical Outcome Study Short Form 36 (SF-36) domain scores at end point (last observation carried forward) for pooled intent-to-treat subjects with *#8805; 50% or < 50% reductions in monthly migraine frequency. *p < 0.001; ^†^*P *= 0.007; ^‡^p = 0.013; ^§^p = 0.019, ^||^p = 0.026 versus < 50% responders. PF = physical functioning; BP = bodily pain; VT = vitality; GH = general health perceptions; RE = role-emotional; RP = role-physical; MH = mental health; SF = social functioning; PCS = physical component summary; MCS = mental component summary.

Significantly greater percentages of patients on topiramate 100 mg/d were ≥ 50% responders versus placebo (46% versus 23%; p < 0.001, Cochran-Mantel-Haenszel test). The individual responder rates for the three pivotal studies were: 54.0% versus 22.6% placebo (p < 0.001) in Silberstein et al. [3]; 49% vs. 23% placebo (p < 0.001) in Brandes et al. [4]; and 37% versus 22% placebo (p < 0.01) in Diener et al. [5]. Baseline MSQ and SF-36 domain scores for those ITT patients who experienced ≥ 50% or < 50% reductions in monthly migraine frequency at end point (last observation carried forward), regardless of study medication, are presented in Table 4; scores were generally similar between the two responder groups. The ≥ 50% responders had significantly higher improvements from baseline to double-blind end point for all three MSQ domains compared with the < 50% responders (Figure 5; p < 0.001). In addition, the ≥ 50% responders had significantly improved SF-36 Physical Component Summary and Mental Component Summary scores and significantly improved scores on seven of eight SF-36 subscales at end point compared with the < 50% responders (Figure 6). Of 262 patients identified as responders, 177 (67.6%) were given topiramate and 85 (32.4%) were on placebo.

**Table 4:** Mean Migraine-Specific Questionnaire (MSQ) and Medical Outcome Study Short Form 36 (SF-36) scores at baseline for pooled intent-to-treat (ITT) subjects with < 50% and ≥ 50% reductions in monthly migraine frequency, regardless of study medication*

	**< 50% Responders**		**≥ 50% Responders**	
	
**MSQ Domain**	**n**	**Baseline**	**n**	**Baseline**
Emotional Function	455	55.5	244	55.2
Role Prevention	454	68.3	243	67.0
Role Restriction	455	50.1	244	49.4
**SF-36 Subscale**	**n**	**Baseline**	**n**	**Baseline**
Physical Functioning	454	83.1	243	80.3
Bodily Pain	453	51.4	244	51.6
Vitality	454	51.7	243	50.0
General Health	447	68.4	242	68.3
Role-Emotional	453	71.0	240	68.2
Role Physical	452	44.6	241	44.1
Mental Health	454	71.3	243	70.7
Social Functioning	455	69.8	244	68.6
Physical Component Score	439	43.2	237	42.8
Mental Component Score	439	47.4	237	46.9

*N values reflect the numbers of patients with evaluable data on each domain, not the ITT population.

## Discussion

Studies detailing the specific impact of migraine preventive therapy on daily activities are limited despite the obvious clinical relevance of such information. The results of this analysis of pooled MSQ and SF-36 data from three pivotal topiramate migraine prevention trials demonstrated that topiramate 100 mg/d was associated with a significant and sustained improvement in daily activities and function for up to 6 months. All MSQ domain scores were significantly improved by week 8 (first health-related quality-of-life assessment point post baseline). The MSQ results indicated that patients on topiramate 100 mg/d had significantly less migraine-related disruption of daily activities and less frustration and feelings of helplessness due to migraine than those on placebo. Topiramate 100 mg/d was also associated with significant improvements in seven of eight subscales of the SF-36. Topiramate-associated improvements on the disease-specific MSQ were stronger than the generic SF-36 scale, which is not surprising given that generic scales tend to measure constructs that are not necessarily affected by a specific disease. In addition, the MSQ does not fully assess the impact of adverse events of drug treatment and may contribute to the higher change scores observed following topiramate treatment.

The Genetic Epidemiology of Migraine study revealed that patient function was inversely related to migraine attack frequency (p < 0.0002), with those suffering from a high frequency of attacks reporting diminished physical, mental, and social functioning [14]. In this analysis, patients who experienced ≥ 50% decreases in monthly migraine frequency on either topiramate or placebo (≥ 50% responders) experienced significantly more improvement in their functioning levels than those patients who experienced < 50% decreases in monthly migraine frequency. This result indicates that reduced monthly migraine frequency is associated with improved patient ability to carry out daily activities, a result supported by earlier clinical trials [6]. Active treatment is superior to placebo, with topiramate 100 mg/d associated with a significantly higher percentage of ≥ 50% responders (46%) than placebo (23%, p < 0.001, Cochran-Mantel-Haenszel test) in a pooled analysis of randomized, double-blind, placebo-controlled trials [6].

The results of this post-hoc study are consistent with pre-specified analyses from two trials that evaluated MSQ and SF-36 outcomes to measure changes in daily activities and function related to topiramate treatment [9,10]. Results of an analysis conducted by Silberstein and colleagues found that in the ITT population (N = 469), topiramate (50 mg/d, 100 mg/d, and 200 mg/d) significantly improved mean MSQ Role Restriction domain scores versus placebo (p = 0.035; p < 0.001; p = 0.001, respectively) [9]. Improvements in mean MSQ Role Prevention scores were significant versus placebo only for topiramate 100 mg/d (p = 0.045). SF-36 Role-Physical and SF-36 Vitality domain scores chosen as outcome measures improved but were not significant versus placebo for topiramate 100 mg/d and 200 mg/d. Changes in these MSQ and SF-36 domain scores significantly correlated with changes in mean monthly migraine frequency.

In an analysis conducted by Brandes and colleagues [10], patients receiving topiramate, 100 or 200 mg/d, had significantly reduced mean monthly migraine frequency (p = 0.008 and p < 0.001, respectively) compared with placebo, but not patients receiving topiramate 50 mg/d (p = 0.48). Topiramate significantly improved mean MSQ Role Restriction domain scores (50 mg/d [p = 0.02], 100 mg/d [p < 0.001], and 200 mg/d [p < 0.001]) and mean MSQ Role Prevention domain scores (50 mg/d [p = 0.007], 100 mg/d [p = 0.001], and 200 mg/d [p = 0.002]) versus placebo. Topiramate 100 and 200 mg/d significantly improved mean SF36 Role-Physical domain scores versus placebo (p = 0.02). Changes in prospectively designated domain scores were significantly correlated with changes in mean monthly migraine frequency (p ≤ 0.001, MSQ domains; p ≤ 0.002, SF-36 domains).

## Conclusion

The results of this study demonstrate that preventive treatment of migraine with topiramate 100 mg/d improves patients' ability to carry out daily activities as early as week 8, and the effect is maintained during 6-month treatment. Information about such patient-centered outcomes is likely to be of interest to migraine patients and clinicians making treatment decisions, but has not been routinely evaluated for most migraine preventive treatments. Reducing the considerable burden and disability of migraine through effective treatment may improve patients' overall functional capacity.

## Abbreviations

ITT = intent-to-treat, MSQ = Migraine-Specific Questionnaire, SF-36 = Medical Outcome Study Short Form 36, PCS = physical component summary, MCS = mental component summary.

## Competing interests

Professor Carl Dahlöf has been a consultant/scientific advisor on advisory boards, clinical trials, and investigator-initiated trials and a speaker for: Allergan, Almirall Prodesfarma, AstraZeneca, Bristol-Myers Squibb, Eisai, GlaxoSmithKline, Janssen Cilag, Merck, Lilly, NMT Medical Inc., Novartis, Ortho-McNeil Pharmaceutical, Pharmacia, Pfizer, Pierre Fabre, and St Jude Medical EMEAC.

Elizabeth Loder has had no financial relationship with any pharmaceutical company since July 2006, except grant support from NMT for a clinical trial. She has been a speaker, received grant support, or been a consultant for: OrthoMcNeil, Endo, AstraZeneca, GlaxoSmithKline, Pfizer, and Allergan. She serves on the Board of Directors of the American Headache Society, the Executive Council of the International Headache Society, and the Board of the Headache Cooperative of New England.

Merle Diamond has served as a consultant and/or conducted research with AstraZeneca, Ortho-McNeil Neurologics, GlaxoSmithKline, Merck and Co., Pfizer, and Primary Care Network.

Marcia Rupnow is a full-time salary employee of Ortho-McNeil Janssen Scientific Affairs, LLC.

George Papadopoulos was an employee of J&J Pharmaceutical Services at the time of study completion.

Lian Mao is a full-time salary employee of Ortho-McNeil Janssen Scientific Affairs, LLC.

## Authors' contributions

All the authors were study investigators and contributed to the development of the manuscript. All authors read and approved the final manuscript.
